# Unraveling the Mysteries of Middle East Respiratory Syndrome Coronavirus

**DOI:** 10.3201/eid2006.140322

**Published:** 2014-06

**Authors:** John T. Watson, Aron J. Hall, Dean D. Erdman, David L Swerdlow, Susan I. Gerber

**Affiliations:** Centers for Disease Control and Prevention, Atlanta, Georgia, USA

**Keywords:** communicable diseases, emerging, coronavirus, viruses, camels, zoonoses, Middle east respiratory syndrome

Middle East respiratory syndrome coronavirus (MERS-CoV) is a novel CoV known to cause severe acute respiratory illness in humans; ≈40% of confirmed cases have been fatal. Human-to-human transmission and multiple outbreaks of respiratory illness have been attributed to MERS-CoV, and severe respiratory illness caused by this virus continues to be identified. MERS-CoV was first reported in September 2012, and subsequent investigations documented illness onsets as early as April 2012 (*1*). As of February 23, 2014, the World Health Organization has reported 182 laboratory-confirmed cases of MERS-CoV infection, including 79 deaths, indicating an ongoing risk for transmission to humans in the Arabian Peninsula (*2*). The median age of reported case-patients is 52 years (range 2–94 years); most case-patients are male (*3*). Most index case-patients have at least 1 reported chronic comorbid condition (*4*) and have resided in, or recently traveled to, Jordan, Qatar, United Arab Emirates, Oman, Kuwait, or Saudi Arabia (*3*). Confirmed cases of MERS-CoV have been identified in travelers returning from France, Germany, Italy, United Kingdom, and Tunisia (*3*). Although some have speculated that a zoonotic reservoir of MERS-CoV exists, very little is known about the specific exposures that result in primary human cases.

MERS-CoV infection causes severe acute hypoxemic respiratory failure, extrapulmonary organ dysfunction, and high rates of death; however, the spectrum of illness and clinical course are not fully defined (*5*). Evidence suggests that MERS-CoV is capable of limited human-to-human transmission, which results in outbreaks in family and health care settings (*5*,*6*). The 182 reported cases include multiple distinct spatiotemporal clusters and 32 identified infections in health care workers (*3*). Modeling performed to assess the extent of human infection and the transmission potential of MERS-CoV (as of August 2013) estimated that most symptomatic case-patients had not been detected but that chains of transmission were not self-sustaining when infection control was implemented (*7*). Despite evidence of human-to-human transmission, the number of contacts infected by persons with confirmed infections appears to be limited; sustained transmission in the community has not been documented (*3*). The Hajj, the annual religious pilgrimage to Saudi Arabia, involved 1.37 million pilgrims from 188 countries in 2013 but resulted in no reports of confirmed cases in the weeks after the pilgrimage (*3*).

Little is known about the pathogenic potential and transmission dynamics of MERS-CoV. Although multiple health care–associated clusters have been identified (*4*), further investigation is needed of the specific risk factors for transmission within health care facilities. Basic information about the temporal and causal patterns of viral shedding and their relationships to clinical outcomes is critical to further understand the virus and to shape prevention and control measures needed to limit transmission. Standard, contact, and airborne precautions appear to be effective in limiting transmission and are recommended by the Centers for Disease Control and Prevention to manage known or suspected MERS-CoV infection in hospitalized patients as a primary means of preventing and controlling transmission (*8*).

Potential animal reservoirs and mechanism(s) of transmission of MERS-CoV to humans remain unclear. Of the minority of case-patients for whom information is available about exposure to animals, few have reported owning or visiting a farm with camels, goats, sheep, chickens, ducks, or other animals (*4*). A zoonotic origin for MERS-CoV was initially suggested by its high genetic similarity to bat CoVs (*9*) and the identification of closely related viruses in bats (*10*). Recent reports have described additional data from camels. These include real-time reverse transcription PCR detection and limited sequencing of MERS-CoV from 3 camels from a farm in Qatar linked to 2 infections in humans in October 2013 (*11*) and, more recently, in camels in Saudi Arabia (*12*), and antibodies against MERS-CoV in serum of camels from the Arabian Peninsula, including serum from the United Arab Emirates drawn in 2003 (*13*–*17*). However, more epidemiologic data linking cases to infected animals are needed to determine whether a particular animal species is a host for the virus, a source of human infection, or both.

This month’s issue of Emerging Infectious Diseases presents results of a study that provides evidence of MERS-CoV in dromedary camels in Egypt (*18*). Only 3 other reports of MERS-CoV detection in animals have been published: 1 in a bat and 2 in camels (*11*,*12*,*19*). However, these reports were based on limited genetic information. In contrast, on the basis of their sequence analysis of nearly the entire viral genome that showed >99% nt sequence identity with human MERS-CoV, Chu et al. provide the most compelling evidence thus far of MERS-CoV infection in dromedary camels (*18*). Although the authors also found neutralizing antibodies to MERS-CoV (or a MERS-like CoV) in most of the camels, they did not find serologic evidence of infection in the abattoir workers who had contact with the infected animals. This finding leaves key questions about zoonotic transmission unanswered. Most notably, it remains unclear whether zoonotic transmission of MERS-CoV occurs between camels and humans and, if so, what the directionality and risk factors are for such transmission. These lingering gaps in knowledge about MERS-CoV emphasize the need for more epidemiologic study to determine risk factors for human infection, more population-level data on the prevalence of MERS-CoV in camels, risk factors for infection and shedding in camels, and continued vigilance for other possible sources of infection. Also, the camels tested were in Egypt and were locally reared or imported from Sudan or Ethiopia, countries in which no cases have been identified in humans. Thus, the geographic area for surveillance should be widened beyond the Arabian Peninsula and include eastern Africa, which is a source for importation of dromedary camels. This study emphasizes the need to further define exposure information for all MERS-CoV cases regarding camels and other animals, as well as exposure to ill humans who might have undetected MERS-CoV infections. Understanding the role of dromedary camels and possibly other animals in transmission of MERS-CoV to humans remains a priority for future investigation to enable development of targeted control measures and prevent future cases and deaths from this emerging pathogen.
